# Supplementary material for: Using performance art to promote intergroup prosociality by cultivating the belief that empathy is unlimited
Source: Nat Commun. 2022 Dec 16;13:7786. doi: 10.1038/s41467-022-35235-z (PMC9756713; doi:10.1038/s41467-022-35235-z)
Supplement: Supplementary file 1 — Supplementary Information [file 41467_2022_35235_MOESM1_ESM.pdf]

Supplementary Information

for

Using performance art to promote intergroup prosociality by cultivating the belief that empathy is unlimited

Yossi Hasson[1,2,4*], Einat Amir[3,4], Danit Sobol-Sarag[2], Maya Tamir[1], Eran Halperin[1]

[1]Psychology Department, The Hebrew University, Mount Scopus, Jerusalem 9190501, Israel; [2]School of Psychology, Reichman University, Herzliya 4610101, Israel; [3]Department of Art and Media & Department of Neuroscience and Biomedical Engineering, Aalto University, FI-00076 Espoo, Finland

[4]Y.H. and E.A. contributed equally to this work.

[*]Corresponding author. Yossi Hasson (yossi.hasson@mail.huji.ac.il)

Supplementary Figure 1. Empathy-inducing articles toward liberals and conservatives

12 People Injured in Liberal Protest

At a liberal protest yesterday in Columbus OH, mass panic resulted in numerous casualties.

By ANDREW TALMADGE

Yesterday around 7 pm, approximately 250 protesters assembled at the meeting point near Nationwide Arena in Columbus, Ohio. Before reaching their final destination at the city hall, the demonstrators had to walk through a narrow pedestrian underpass.

For reasons yet unknown, panic began to spread. While dozens of protesters desperately tried to escape from the crowd, people from behind kept pushing forward – unaware of the tragedy that was unfolding ahead.

“It was awful”, recounts an eyewitness. “I was afraid for my life. People were so desperate, crying for help and we just couldn’t get out.” In total, 12 people were injured.

Medics arriving at the scene were faced with extensive bruises and head injuries. Some of the protesters were even taken to a hospital for observation and additional medical treatment.

12 People Injured in Conservative Protest

At a conservative protest yesterday in Columbus OH, mass panic resulted in numerous casualties.

By ANDREW TALMADGE

Yesterday around 7 pm, approximately 250 protesters assembled at the meeting point near Nationwide Arena in Columbus, Ohio. Before reaching their final destination at the city hall, the demonstrators had to walk through a narrow pedestrian underpass.

For reasons yet unknown, panic began to spread. While dozens of protesters desperately tried to escape from the crowd, people from behind kept pushing forward – unaware of the tragedy that was unfolding ahead.

“It was awful”, recounts an eyewitness. “I was afraid for my life. People were so desperate, crying for help and we just couldn’t get out.” In total, 12 people were injured.

Medics arriving at the scene were faced with extensive bruises and head injuries. Some of the protesters were even taken to a hospital for observation and additional medical treatment.

Supplementary Figure 2. Manipulation of the belief about empathy as a limited/unlimited resource

Definition of empathy (presented in both conditions – limited and unlimited empathy)

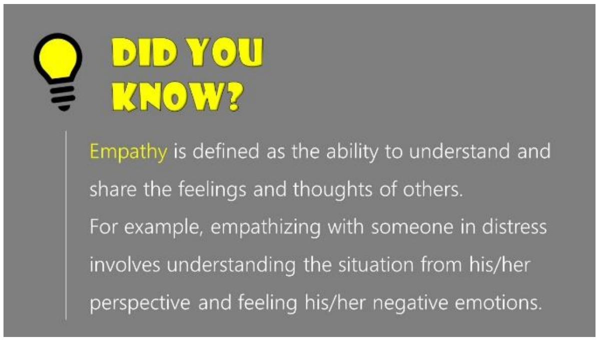

Limited empathy condition

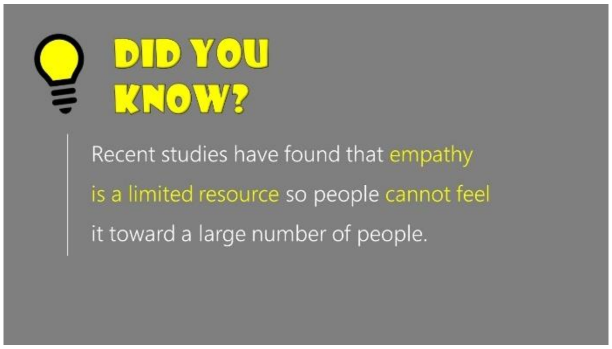

Imagine that you are about to meet people in distress. Toward how many of them could you feel empathy?

Please answer by using the scale below:
From 0 - can't feel empathy toward anyone; To 3 - can feel empathy toward three people.

0 1 2 3

number of people I can empathize with

Unlimited empathy condition

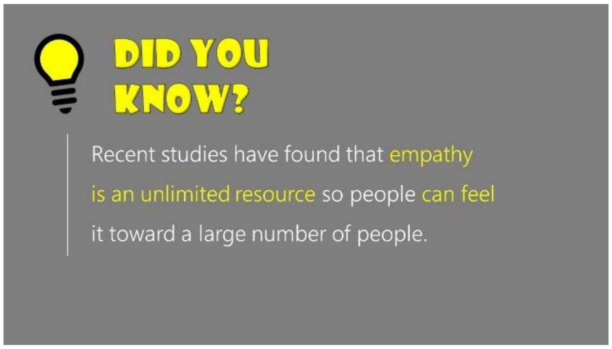

Imagine that you are about to meet people in distress. Toward how many of them could you feel empathy?

Please answer by using the scale below:
From 0 - can't feel empathy toward anyone; To 300 - can feel empathy toward three hundred people.

0 30 60 90 120 150 180 210 240 270 300

number of people I can empathize with

Supplementary Figure 3. Manipulation of the belief about empathy as a limited/unlimited resource or control

Control condition (definition of empathy)

"Empathy is defined as the ability to understand and share the feelings of others. For example, empathizing with someone in distress involves understanding the situation from his/her perspective and feeling his/her negative emotions"

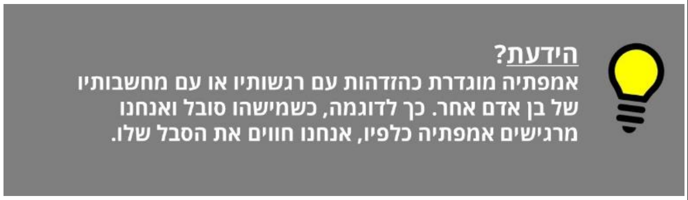

Unlimited empathy condition (definition of empathy + unlimited empathy text)

"Apparently the idiom 'everyone has a place in my heart' is scientifically correct: Studies have found that empathy is an unlimited resource so it can be felt toward a large number of people"

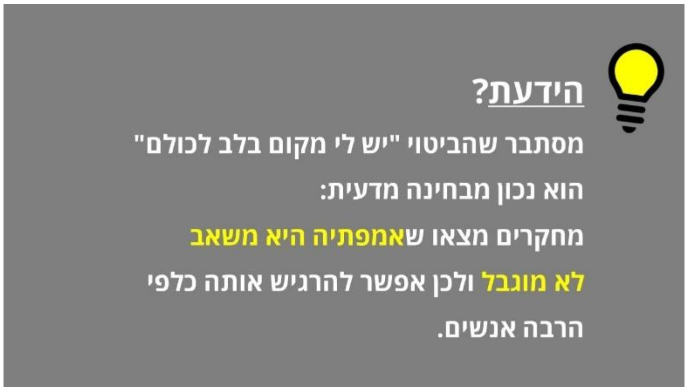

Limited empathy condition (definition of empathy + limited empathy text)

"Apparently the idiom 'everyone has a place in my heart' is scientifically incorrect: Studies have found that empathy is a limited resource so it cannot be felt toward a large number of people"

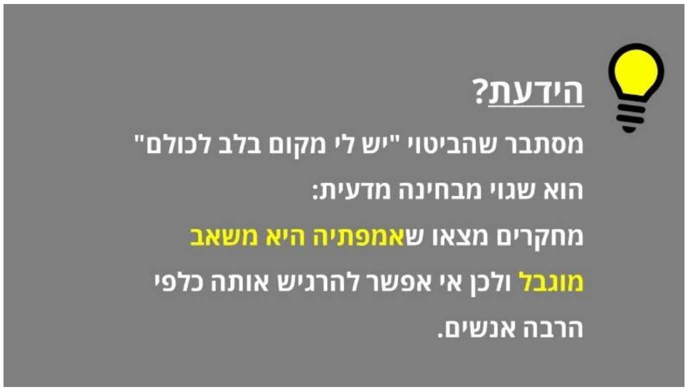

Supplementary Figure 4. Empathy-inducing articles toward Jews and Arabs. Type of issue (financial and health problems) and Group identity (Jews and Arabs) were counter-balanced.

<table>
<tr>
<td>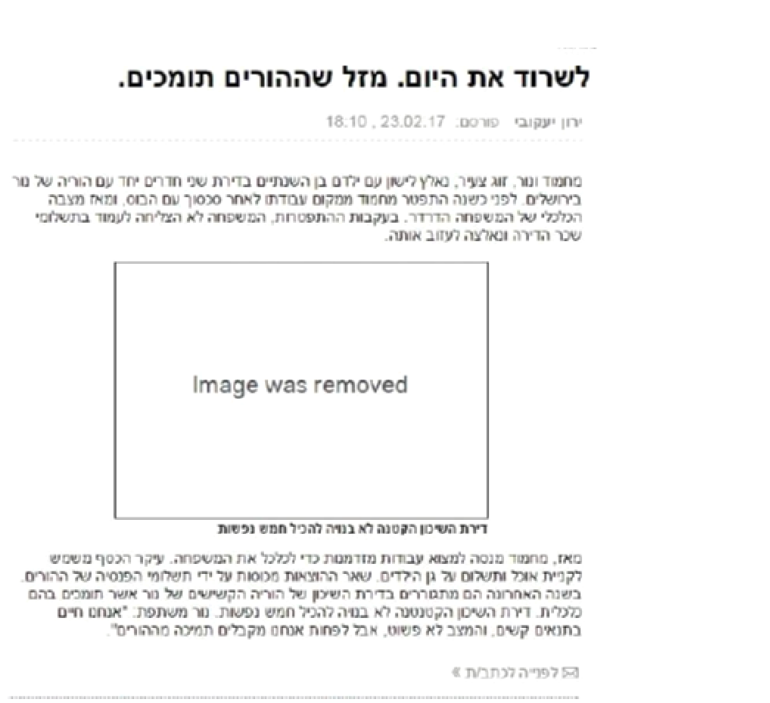
לשרוד את היום. מזל שההורים תומכים.
ירון יעקובי פורסם: 23.02.17 , 18:10
מחמוד ונור, זוג צעיר, נאלץ לישון עם ילדם בן השנתיים בדירת שני חדרים יחד עם הוריה של נור בירושלים. לפני כשנה התפטר מחמוד ממקום עבודתו לאחר סכסוך עם הבוס, ומאז מצבה הכלכלי של המשפחה הדרדר. בעקבות ההתפטרות, המשפחה לא הצליחה לעמוד בתשלומי שכר הדירה ונאלצה לעזוב אותה.
Image was removed
דירת השיכון הקטנה לא בנויה להכיל חמש נפשות
מאז, מחמוד מנסה למצוא עבודות מזדמנות כדי לכלכל את המשפחה. עיקר הכסף משמש לקניית אוכל ותשלום על גן הילדים. שאר ההוצאות מכוסות על ידי תשלומי הפנסיה של ההורים. בשנה האחרונה הם מתגוררים בדירת השיכון של הוריה הקשישים של נור אשר תומכים בהם כלכלית. דירת השיכון הקטנטנה לא בנויה להכיל חמש נפשות. נור משתפת: "אנחנו חיים בתנאים קשים, והמצב לא פשוט, אבל לפחות אנחנו מקבלים תמיכה מההורים".
לפנייה לכתב/ת »</td>
<td>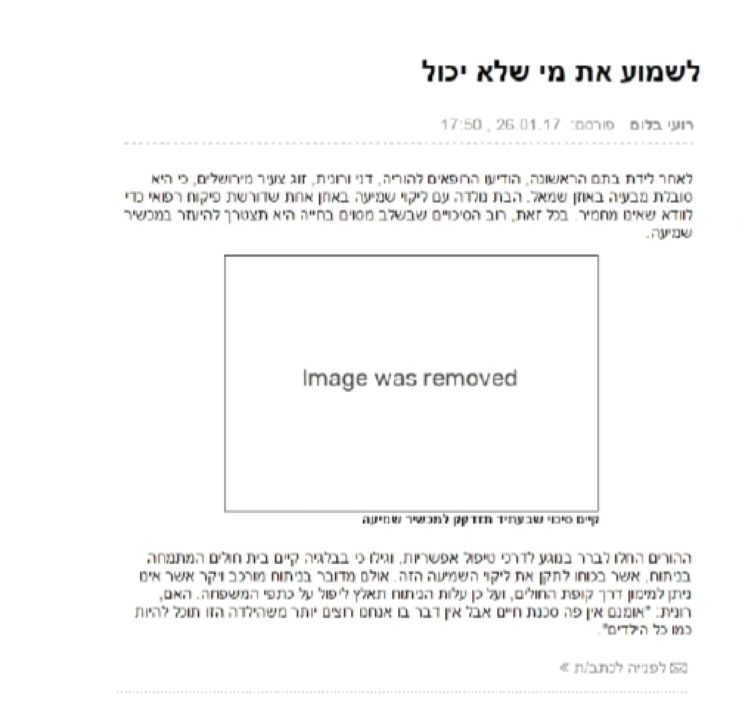
לשמוע את מי שלא יכול
רועי בלום פורסם: 26.01.17 , 17:50
לאחר לידת בתם הראשונה, הודיעו הרופאים להוריה, דני ורונית, זוג צעיר מירושלים, כי היא סובלת מבעיה באוזן שמאל. הבת נולדה עם ליקוי שמיעה באוזן אחת שדורשת פיקוח רפואי כדי לוודא שאינו מחמיר. בכל זאת, רוב הסיכויים שבשלב מסוים בחייה היא תצטרך להיעזר במכשיר שמיעה.
Image was removed
קיים סיכוי שבעתיד תזדקק למכשיר שמיעה
ההורים החלו לברר בנוגע לדרכי טיפול אפשריות, וגילו כי בבלגיה קיים בית חולים המתמחה בניתוח, אשר בכוחו לתקן את ליקוי השמיעה הזה. אולם מדובר בניתוח מורכב ויקר אשר אינו ניתן למימון דרך קופת החולים, ועל כן עלות הניתוח תאלץ ליפול על כתפי המשפחה. האם, רונית: "אומנם אין פה סכנת חיים אבל אין דבר בו אנחנו רוצים יותר משהילדה הזו תוכל להיות כמו כל הילדים".
לפנייה לכתב/ת »</td>
</tr>
<tr>
<td>SURVIVE THE DAY. LUCKY PARENTS SUPPORT
Muhammad and Nur, a young couple, have no other choice but to sleep with their two-year-old child in a two-room apartment with Nur's parents in Jerusalem. About a year ago, Muhammad resigned from his job after a dispute with his manager, and since then the family's financial situation has deteriorated. Following the resignation, the family was unable to meet the rent payments and was forced to leave the apartment.
Since then, Muhammad has been trying to find casual jobs to support the family. Most of the money is used to buy food and pay for the kindergarten. The rest of the expenses are covered by the parents' pension payments. For the past year they have been living in the apartment of Nur's</td>
<td>HEAR THOSE WHO CANNOT
After the birth of their first daughter, the doctors informed her parents, Dani and Ronit, a young couple from Jerusalem, that she was suffering from a problem in her left ear. The daughter was born with a hearing loss in one ear that requires medical supervision to make sure it does not become worse. However, chances are that at some point in her life she will need to use a hearing aid.
The parents started looking for possible treatments and found that in Belgium there is a hospital that specializes in surgeries that can fix this hearing impairment. However, this is a complex and expensive operation that cannot be financed through their Health maintenance</td>
</tr>
</table>

elderly parents who support them financially. The tiny apartment is not planned for accommodating five people. Nur shares: "We live in difficult conditions, and the situation is not simple, but at least we get support from the parents."	organization, and therefore the cost of the operation will have to fall on the family’s shoulders. The mother, Ronit: "Although this is not a life-threatening condition, what we want most is that our girl would be like all our children".
