# Supplementary material for: Using performance art to promote intergroup prosociality by cultivating the belief that empathy is unlimited
Source: Nat Commun. 2022 Dec 16;13:7786. doi: 10.1038/s41467-022-35235-z (PMC9756713; doi:10.1038/s41467-022-35235-z)
Supplement: Supplementary file 3 — Reporting Summary [file 41467_2022_35235_MOESM3_ESM.pdf]

nature portfolio

Corresponding author(s): Yossi Hasson

Last updated by author(s): Jun 4, 2022

Reporting Summary

Nature Portfolio wishes to improve the reproducibility of the work that we publish. This form provides structure for consistency and transparency in reporting. For further information on Nature Portfolio policies, see our Editorial Policies and the Editorial Policy Checklist.

Statistics

For all statistical analyses, confirm that the following items are present in the figure legend, table legend, main text, or Methods section.

n/a	Confirmed	
☐	☒	The exact sample size (*n*) for each experimental group/condition, given as a discrete number and unit of measurement
☐	☒	A statement on whether measurements were taken from distinct samples or whether the same sample was measured repeatedly
☐	☒	The statistical test(s) used AND whether they are one- or two-sided<br>*Only common tests should be described solely by name; describe more complex techniques in the Methods section.*
☐	☒	A description of all covariates tested
☐	☒	A description of any assumptions or corrections, such as tests of normality and adjustment for multiple comparisons
☐	☒	A full description of the statistical parameters including central tendency (e.g. means) or other basic estimates (e.g. regression coefficient) AND variation (e.g. standard deviation) or associated estimates of uncertainty (e.g. confidence intervals)
☐	☒	For null hypothesis testing, the test statistic (e.g. *F*, *t*, *r*) with confidence intervals, effect sizes, degrees of freedom and *P* value noted<br>*Give P values as exact values whenever suitable.*
☒	☐	For Bayesian analysis, information on the choice of priors and Markov chain Monte Carlo settings
☒	☐	For hierarchical and complex designs, identification of the appropriate level for tests and full reporting of outcomes
☐	☒	Estimates of effect sizes (e.g. Cohen's *d*, Pearson's *r*), indicating how they were calculated

Our web collection on statistics for biologists contains articles on many of the points above.

Software and code

Policy information about availability of computer code

Data collection	Data was collected via Qualtrics v.2016, 2017, 2018
Data analysis	Data was analyzed by SPSS v.25

For manuscripts utilizing custom algorithms or software that are central to the research but not yet described in published literature, software must be made available to editors and reviewers. We strongly encourage code deposition in a community repository (e.g. GitHub). See the Nature Portfolio guidelines for submitting code & software for further information.

Data

Policy information about availability of data

All manuscripts must include a data availability statement. This statement should provide the following information, where applicable:

- Accession codes, unique identifiers, or web links for publicly available datasets
- A description of any restrictions on data availability
- For clinical datasets or third party data, please ensure that the statement adheres to our policy

All data analyzed during the current studies are available at Open Science Framework, https://osf.io/4u26s 83. Source data are provided with this paper.

Field-specific reporting

Please select the one below that is the best fit for your research. If you are not sure, read the appropriate sections before making your selection.

☐ Life sciences ☒ Behavioural & social sciences ☐ Ecological, evolutionary & environmental sciences

For a reference copy of the document with all sections, see nature.com/documents/nr-reporting-summary-flat.pdf

Behavioural & social sciences study design

All studies must disclose on these points even when the disclosure is negative.

Study description

All data collected in the studies is quantitative and includes self-report and behavioral measures.

Research sample

Pilot study: A sample of 1308 Israeli teenagers (Mage = 16.65 years, SD = 2.66; 53.7% females) from three different social groups in Israel – Jewish secular, Jewish religious, and Arab. Data was collected as part of a nationwide education project via local survey companies.

Study 1: To obtain 80% power for a moderation model, we needed a sample size of 100 participants based on the lowest effect size found in the pilot study (d=.2). Given the study aimed to examine an additional hypothesis (unrelated to the current project) that required more participants, the final sample was almost doubled. A sample of 182 U.S. participants (Mage = 34.56 years, SD = 11.29, 57.7% females). Data was collected via Amazon's Mechanical Turk.

Study 2: To obtain 80% power for a mixed design with both within- and between-participants factors, we needed a sample size of 194 participants based on the lowest effect size found in the pilot study (d=.2). We slightly over-sampled to account for possible attrition. A sample of 200 U.S. participants (Mage = 33.5 years, SD = 11.5, 57.5% females). Data was collected via Amazon's Mechanical Turk.

Study 3: A sample of 150 Israeli-Jewish participants (Mage = 37.33 years, SD = 12.97, 52.7% females). Data was collected via a local survey company. The sample size was determined based on the effect size found among the Jewish sample in the pilot study (d=.37). To obtain 80% power we needed a sample size of 144 participants. We slightly over-sampled to account for possible attrition.

Study 4: A priori power analyses based on the effect size of empathic reactions in Study 2 lead to unacceptably small sample sizes (i.e., less than 15 participants). Therefore, we aimed to recruit at least 100 participants (50 in each of the two conditions). A sample of 108 American participants (Mage = 34.8 years, SD = 11.5, 55.6% females) who attended a performance art-experiment that took place in a performance venue in Chicago, USA.

Study 5: A priori power analyses based on the effect size of empathic reactions in Study 3 led to a very small sample size (i.e., less than 50 participants). Therefore, we aimed to recruit at least 100 participants (50 in each of the two conditions). However, given that the study was part of an open perforce art, we accepted more participants to take part in the event. A sample of 176 Israeli-Jewish participants (Mage = 26.84 years, SD = 13.89, 50.3% females) who attended a performance art-experiment that took place in a venue in Jerusalem, Israel.
Samples were chosen from social contexts that involve intergroup tensions including ethnic, national, religious, and political ones.

Sampling strategy

Sample sizes of the studies were determined as follows:
- Pilot Study: The sample was determined based on considerations related to additional hypotheses of a nationwide education project. While a representative sample of the Israeli population requires a sample of ~500 responses, our sample included 1308 participants. Random sampling.
- Study 1: To obtain 80% power for a moderation model, we needed a sample size of 100 participants based on the lowest effect size found in the pilot study (d=.2). Given the study aimed to examine additional hypothesis that required more participants the final sample was almost doubled. Random sampling.
- Study 2: To obtain 80% power for a mixed design with both within- and between-participants factors, we needed a sample size of 194 participants based on the lowest effect size found in the pilot study (d=.2). We slightly over-sampled to account for possible attrition. Random sampling.
- Study 3: The sample size was determined based on the effect size found among the Jewish sample in the pilot study (d=.37). To obtain 80% power we needed a sample size of 144 participants. We slightly over-sampled to account for possible attrition. Random sampling.
- Study 4: A priori power analysis based on Study 2 lead to unacceptably small sample sizes (i.e., less than 15 participants). Therefore, we aimed to recruit at least 100 participants (50 in each of the two conditions). Convenience sampling.
- Study 5: A priori power analysis based on Study 3 led to a very small sample size (i.e., less than 50 participants). Therefore, we aimed to recruit at least 100 participants (50 in each of the two conditions) but given it was part of an open perforce art we continue collecting during all the event. Convenience sampling.

Data collection

Pilot study and Studies 1-3 were conducted online via desktop or mobile devices.
Studies 4 & 5 were conducted as part of performance art-experiment events in which participants met an actor face-to-face. During data collection the actors who collected the data were blind to the experimental conditions and the hypotheses of the studies. Data was collected via tablets.

Timing

The six studies were conducted between 2016-2018 in the following time periods:
- Feb 11, 2016 – April 10, 2016
- Aug 9-14, 2017

	• Aug 24-25, 2017<br>• Dec 12, 2017 – March 17, 2018<br>• April 28-29, 2018<br>• May 11-13, 2018
Data exclusions	In Study 5 two participants were not included in the analysis of empathic feelings because they did not rate it due to technical issues during the performance-experiment. Other data related to these participants was analyzed.
Non-participation	In Study 5 two participants (out of 176; 1.14%) chose not to participate in the performance-experiment after being assigned to the control condition.
Randomization	In all experimental studies, participants were randomly assigned into experimental groups.

Reporting for specific materials, systems and methods

We require information from authors about some types of materials, experimental systems and methods used in many studies. Here, indicate whether each material, system or method listed is relevant to your study. If you are not sure if a list item applies to your research, read the appropriate section before selecting a response.

Materials & experimental systems

n/a	Involved in the study
☒	☐ Antibodies
☒	☐ Eukaryotic cell lines
☒	☐ Palaeontology and archaeology
☒	☐ Animals and other organisms
☐	☒ Human research participants
☒	☐ Clinical data
☒	☐ Dual use research of concern

Methods

n/a	Involved in the study
☒	☐ ChIP-seq
☒	☐ Flow cytometry
☒	☐ MRI-based neuroimaging

Human research participants

Policy information about studies involving human research participants

Population characteristics	Please see above
Recruitment	In the pilot study and Studies 1-3 participants were recruited via survey companies or Amazon's Mechanical Turk.<br>In Studies 4 & 5 participants were registered in advance to the performance-experiments. To avoid potential self-selection bias, the advertisement and description of the events did not include any information related to the topic of the study (e.g., empathy, intergroup relations).
Ethics oversight	The Institutional Review Board (IRB) at Reichman University (former name: the Interdisciplinary Center), Herzliya

Note that full information on the approval of the study protocol must also be provided in the manuscript.
